# FedeAMR-CFF: A Federated Automatic Modulation Recognition Method Based on Characteristic Feature Fine-Tuning

**DOI:** 10.3390/s25134000

**Published:** 2025-06-26

**Authors:** Meng Zhang, Jiankun Ma, Zhenxi Zhang, Feng Zhou

**Affiliations:** 1 Southwest China Institute of Electronic Technology, Chengdu 610036, China; alice_mm@163.com; 2 Key Laboratory of Electronic Information Counter-Measure and Simulation, Ministry of Education, Xidian University, Xi’an 710071, China; 23021211464@stu.xidian.edu.cn; 3 School of Aerospace Space and Technology, Xidian University, Xi’an 710071, China; fzhou@mail.xidian.edu.cn

**Keywords:** automatic modulation recognition, federated learning, fine-tuning

## Abstract

Modulation recognition technology, as one of the core technologies in the field of wireless communications, holds significant importance in intelligent communication systems such as link adaptation and IoT devices. In recent years, deep learning-based automatic modulation recognition (DL-AMR) has emerged as a major research direction in this domain. Existing DL-AMR schemes primarily adopt a centralized training architecture, where a unified model is trained on a central server using local data from terminal devices. Although such methods achieve high recognition accuracy, they carry substantial privacy leakage risks. Moreover, when terminal devices independently train models solely based on their local data, the model performance often suffers due to issues like data distribution disparities and insufficient training samples. To address the critical challenges of high data privacy leakage risks, excessive communication overhead, and data silos in automatic modulation recognition tasks, this paper proposes a federated automatic modulation recognition method based on characteristic feature fine-tuning (FedeAMR-CFF). Specifically, the clients extract representative features through distance-based metric screening, and the server aggregates model parameters via the FedAvg algorithm and fine-tunes the model using the collected features. This method not only safeguards client data privacy but also facilitates effective knowledge transfer across distributed datasets while significantly mitigating the non-independent and identically distributed problem. Experimental validation demonstrates that FedeAMR-CFF achieves an improvement of 3.43% compared to the best-performing local model.

## 1. Introduction

Automatic modulation recognition (AMR) plays a vital role in modern wireless communication systems by enabling intelligent receivers to recognize modulation schemes without prior knowledge, which is essential for spectrum management, cognitive radio, and military applications [1,2]. Based on methodological differences, existing AMR methods [3] can be primarily categorized into two classes: discriminative approaches based on feature extraction and generative approaches based on probabilistic models [4,5]. The former mainly relies on feature engineering constructed from expert knowledge (e.g., higher-order statistics and spectral features) combined with traditional machine learning classifiers (e.g., support vector machines (SVMs) [6] and decision trees [7]). The latter achieves classification through likelihood function construction, but its performance is severely constrained by the requirement for precise prior knowledge of channel parameters (e.g., phase offset and noise power).

Recent breakthroughs in deep learning (DL) technology within fields such as computer vision [8] and natural language processing [9] have established a new paradigm [10] for modulation recognition research [11]. DL-based AMR methods have demonstrated superior classification performance in complex electromagnetic environments. Currently, mainstream solutions predominantly adopt centralized learning architectures, where edge nodes upload raw signal data to central servers for unified modeling [12,13,14]. However, this paradigm suffers from two inherent limitations: firstly, the transmission of raw signals containing sensitive user information poses potential privacy leakage risks that may violate data protection regulations [15]. Secondly, when relying solely on local data for model training, the limited data volume often results in model performance that fails to meet practical requirements.

Federated learning (FL) effectively addresses the aforementioned contradiction through its innovative data stays while models move paradigm. This framework replaces raw data centralization with distributed model parameter aggregation, achieving collaborative modeling while ensuring data privacy [16]. The emergence of federated AMR [17] introduces a transformative approach that addresses critical challenges in privacy, security, and scalability while maintaining high recognition accuracy. Unlike traditional centralized AMR methods, which require raw data aggregation and pose significant privacy risks, federated AMR allows multiple devices or edge nodes to collaboratively train a shared model without exposing local data, thereby preserving user confidentiality and complying with stringent data protection regulations. This distributed framework also enhances security by mitigating single-point failures and adversarial attacks, making it particularly valuable in defense and secure communications. Moreover, federated AMR improves scalability and efficiency in large-scale networks like IoT and 5G/6G by reducing latency and bandwidth overhead, as data processing occurs locally rather than in a centralized server. The ability to learn from diverse, distributed datasets further enhances adaptability to dynamic channel conditions, such as multipath fading and noise variations, leading to more robust performance in real-world environments. Despite these advantages, challenges such as non-IID data distribution and synchronization delays require further research to optimize communication efficiency and model aggregation strategies. By integrating federated learning with AMR, this approach not only advances wireless intelligence but also aligns with the growing demand for privacy-aware, decentralized AI solutions, paving the way for next-generation secure and adaptive communication systems [18].

Specifically, every client device undertakes model training utilizing its private data, exclusively forwarding the parameter updates to be merged at the server level. The generated global model is then redistributed to all terminals. However, the standard FL framework is built upon an idealized “client homogeneity” assumption, presupposing all participants share similar data distributions and computational capabilities [19]. In practical communication scenarios, the “data heterogeneity” issue caused by device diversity and channel environment variations significantly degrades the generalization performance of global models.

To address these challenges, this paper innovatively proposes a federate automatic modulation recognition network, namely FedeAMR-CFF. The framework achieves the synergistic improvement of privacy protection and model performance through a dual-optimization mechanism: at the client level, a distance metric-based feature selection algorithm extracts the most representative feature vectors from each modulation category. At the server level, it first aggregates global model parameters via the federated averaging (FedAvg) algorithm, then performs targeted fine-tuning on the fully connected classification layer using collected characteristic features. This design facilitates knowledge transfer through characteristic features rather than raw data sharing, effectively alleviating the prevalent non-independent and identically distributed (non-IID) issue in FL while strictly maintaining data privacy. The main innovations of this work can be summarized in three aspects:

- To highlight the more effective federate learning in AMR, this paper proposes FedeAMR-CFF, an innovative federated automatic modulation recognition framework that uniquely integrates parameter aggregation with characteristic feature fine-tuning.
- To address the data privacy issue, we propose a distance metric-based feature selection method at the client level to extract the most representative features of each modulation type.
- To address the distribution discrepancy issue, we propose a targeted fine-tuning method on the classification layer at the server level using the selected representative features based on FedAvg, significantly enhancing the generalization capability across clients with heterogeneous data distributions.
- Extensive experiments on the RML2016.10A dataset validate our model’s superior performance, indicating that FedAMR-CFF outperforms the best local model by 3.43% in recognition accuracy.

## 2. Related Work

### 2.1. Automatic Modulation Recognition

Research methodologies for AMR can be primarily categorized into two classes: probability model-based (LB-AMR) and feature extraction-based (FB-AMR) approaches [20,21,22]. The LB-AMR methods construct likelihood functions based on Bayesian theory, which can theoretically achieve optimal recognition performance but suffer from high computational complexity and stringent requirements for precise channel parameter estimation [20,21]. The FB-AMR methods employ transform-domain features [23] and the statistical features [24] of signals integrated with conventional machine learning approaches, including support vector machines and decision tree classifiers. However, existing traditional methods exhibit certain limitations, underscoring the critical demand for developing specific techniques to achieve optimal feature representation. Their performance heavily relies on the quality of manually designed features [22].

As deeper neural architectures have generally yielded better training results in recent years, O’Shea et al. [25] used deep networks with raw IQ input data for end-to-end modulation recognition. Peng et al. [26] employed constellation diagram transformation combined with AlexNet and GoogLeNet for classification. A residual network [27] was constructed, with multiple convolutional layers stacked together for AMR. In an effort to capture temporal and spatial relationships at different scales within signals, a cost-effective CNN [28] was developed. This CNN design incorporated parallel arrangements of convolutional kernels with diverse sizes, enabling it to effectively model the multi-scale features of the input data. Tan et al. [29] proposed a few-shot automatic modulation classification framework, termed Multi-Scale Fusion and Distribution Similarity Network, for rapid adaptation to new tasks with few-shot training samples. A hierarchical feature extractor that captures contextual signal patterns across diverse receptive fields was designed to enhance discriminative representation learning. Tan et al. [3] formulated a few-shot class-incremental AMC (FSCI-AMC) problem and developed a pseudo-class stochastic classifier network for this task. The framework first creates pseudo-classes to preserve the capacity for novel modulation types, improving continual learning performance. Stochastic classifiers maintain pseudo-class reliability during generation. During incremental phases, the system jointly utilizes both authentic and pseudo-classes for classification.

In addition to CNNs, recurrent neural networks (RNNs) have been applied to AMR. West et al. [30] designed a CLDNN network that combines the advantages of CNN and LSTM, improving feature reuse efficiency through cross-layer connections. Xu et al. [31] proposed a three-stream convolutional long short-term deep neural network. It extracts more abundant modulation information via independent I/Q multichannels and mitigates the misclassification issue between 16QAM and 64QAM. Traditional neural networks often struggle with long-sequence dependencies, but the Transformer architecture offers a solution [32,33]. Owing to its self-attention mechanism, Transformer facilitates efficient parallel processing and is adept at extracting comprehensive global features from signal sequences, providing a more holistic understanding of the input signal. Cai et al. converted IQ sequences into image patches and utilized Vision Transformer for global feature modeling [34]. Zhai et al. [35] proposed a multimodal Transformer-based architecture, integrating wavelet transform and a power spectrum to extract cross-domain signal features.

Although these methods have achieved remarkable results, they do not adequately account for deployment in distributed environments nor address potential user privacy breaches during data transmission. Therefore, the study of FL can offer three main advantages for radio signal processing systems. By keeping raw data localized and only transmitting model parameters or gradients, FL inherently preserves data privacy while maintaining the utility of distributed datasets. This framework substantially reduces communication overhead compared to traditional raw signal transmission, as parameter updates require significantly lower bandwidth consumption. Furthermore, FL enables cross-domain collaboration without data sharing, effectively breaking down information silos and facilitating joint model optimization across heterogeneous devices. This cooperative paradigm not only enhances overall recognition accuracy but also improves generalization capabilities through diversified yet privacy-preserving training.

### 2.2. Federated Learning

The conventional cloud-based deep learning paradigm typically requires transmitting data collected by edge devices to central servers for centralized processing and model training. However, with increasingly stringent data privacy protection regulations, directly transferring raw edge data not only presents technical challenges but may also violate relevant laws and regulations. To address this dilemma, FL has emerged as a distributed collaborative mechanism that enables multi-device joint modeling while ensuring user data remains localized on edge devices [36,37].

As an innovative privacy-preserving distributed learning paradigm, FL adopts a core philosophy of moving models instead of data, presenting a stark contrast to traditional centralized training approaches. Specifically, FL performs model training on local devices and only uploads parameter updates rather than sharing raw data, thereby effectively addressing data privacy leakage issues [36]. This decentralized learning approach not only breaks down “data silos” but also significantly enhances the security and compliance of model training. Currently, FL technology has demonstrated substantial application value across multiple critical domains, including healthcare [38] and wireless communications [39].

In the field of wireless communications, FL technology has been primarily applied to resource optimization problems. For vehicular networks with ultra-reliable low-latency communication requirements, Samarakoon et al. [39] developed an FL-based tail distribution estimation algorithm for queue lengths, achieving joint optimization of resource allocation and power control. The experimental results demonstrate that this approach maintains comparable performance to centralized methods while offering superior privacy preservation. The research team, led by Chen [40], further proposed an FL-based framework for joint user selection and resource allocation, providing novel insights for 5G network resource management.

At the algorithmic implementation level, McMahan et al. [41] proposed the FedAvg algorithm, which reduces communication frequency by increasing the number of local stochastic gradient descent (SGD) iterations. Compared to the baseline FedSGD algorithm, FedAvg achieves an approximate 10% reduction in communication overhead while maintaining similar model recognition accuracy. However, this algorithm lacks theoretical convergence guarantees for non-convex optimization problems and demonstrates suboptimal performance in non-IID data scenarios. To address these challenges, our approach employs a distance-metric-based feature selection algorithm to extract representative feature vectors from each modulation category, which are then utilized for model fine-tuning to mitigate non-IID data issues.

## 3. Methodology

**Figure 1:**
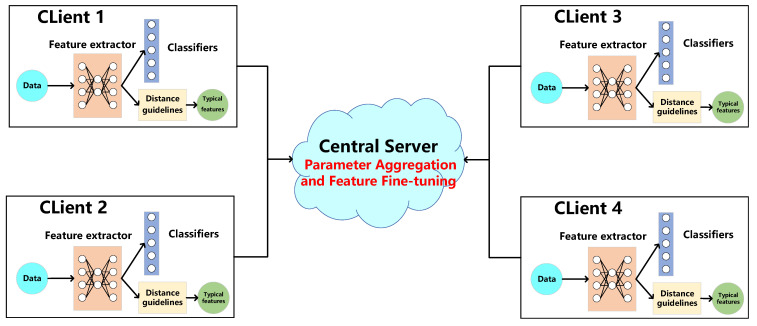
The scheme of FedeAMR-CFF: During local training at each client, characteristic feature vectors are selected for every modulation type, and both the model parameters and these characteristic features are transmitted to the central server for aggregated fine-tuning.

In this section, we provide a detailed introduction to FedeAMR-CFF, as illustrated in Figure 1. We first introduce the signal model and dataset in Section 3.1, followed by the model architecture we adopted in Section 3.2 and the model aggregation method in Section 3.3. Then, we elaborate on the proposed distance-based typical feature selection method in Section 3.4. Finally, we describe the feature fine-tuning method in Section 3.5.

### 3.1. Signal Model and Dataset

In this paper, the received signal is represented as follows:

(1) $$x_{r}\left(n\right)=A\left(n\right)e^{j\left(\theta_{0}+2\pi fn\right)}x_{t}\left(n\right)+w\left(n\right),\;n\in \left[0,L-1\right]$$

where *L* is the sequence length, and *n* denotes the index of a sequence. $x_{r}\left(n\right)$, $x_{t}\left(n\right)$, and w(n) denote the received signal, the transmitted modulation signal, and the additive white Gaussian noise (AWGN), respectively. Additionally, the time-varying channel gain A(n) follows a Rayleigh distribution with its value range bounded within [0,+∞). $\theta_{0}$ and *f* are the frequency offset and phase offset, respectively, and *L* represents the signal length.

This study adopts the widely recognized RML2016.10A benchmark dataset [12], which serves as a standard evaluation tool in radio signal processing research. The dataset is synthesized through professional equipment with real-world semantic sources. The digital modulation signals are generated from Shakespeare’s Gutenberg Project texts, while analog modulation signals originate from the podcast series ’Serial Episode’. These baseband signals are processed through high-fidelity channel modeling using GNU Radio. The adopted propagation channel framework integrates additive white Gaussian noise (AWGN) along with multipath propagation effects. This comprehensive channel modeling approach accounts for multiple random impairments, including clock synchronization errors, carrier frequency discrepancies, and time-varying attenuation characteristics, to accurately emulate practical operational conditions. These introduced distortion factors enable the dataset to effectively emulate the characteristic features of real-world wireless communication environments.

RML2016.10A comprises 220,000 modulated signals with SNR ranging from −20 dB to 18 dB. Each sample has a signal length of 128, covering 11 modulation types: 16-QAM, 4-PAM, 64-QAM, WBFM, CPFSK, AM-SSB, BPSK, GFSK, AM-DSB, QPSK, and 8-PSK. In the experimental setup, we utilized all available data from RML2016.10A and divided the dataset into training, validation, and test sets in a 7:1:2 ratio. The training and validation sets were further randomly partitioned into four equal parts to serve as the local data for each client.

### 3.2. Multi-Channel Temporal Feature Extraction

The edge-side network employs an MCLDNN architecture [31], which consists of three core components: multi-channel feature extraction, temporal feature modeling, and a fully connected classifier. I/Q samples constitute the most basic physical-layer signal representation in digital receivers, containing complete waveform information from which all other features, including A/P features, can be derived. This makes I/Q features the most versatile foundation for federated learning.

**Figure 2:**
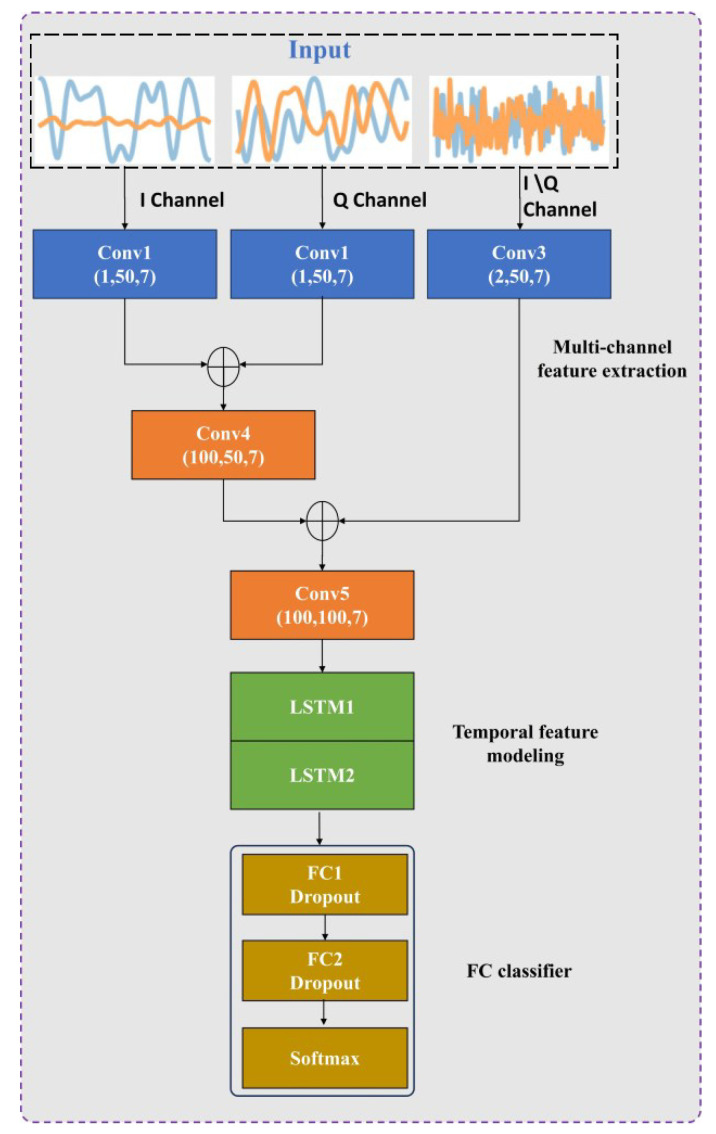
MCLDNN network structure.

As illustrated in Figure 2, the processing pipeline initiates with three parallel 1D convolutional neural networks (conv1, conv2, and conv3) operating on distinct input modalities, the I-channel, Q-channel, and combined I/Q-channel signals, respectively. The server side then merges the I/Q feature streams through a concatenation operation, first combining the I-channel and Q-channel outputs via conv4, then fusing this combined representation with the I/Q-channel features to form a comprehensive multi-channel feature representation.

The temporal modeling stage processes these integrated features through conv5, followed by dual LSTM networks with a 128-cell architecture for sequential pattern recognition. These recurrent modules capture long-range temporal dependencies in the signal characteristics. The final classification stage employs a two-layer, fully connected network terminated with Softmax activation, providing modulation-type prediction capability.

### 3.3. Model Aggregation Method

The FedAvg algorithm is widely used in federated learning to aggregate model updates from multiple local clients without transferring raw data. Below is a detailed breakdown of the steps involved in training a central modulation recognition model using FedAvg, including the update procedure for local models.

Step 1: Initialize Global Model

The central server initializes a global model $\theta_{0}$, which is shared across all clients. The model is typically initialized with random weights or pre-trained values based on prior knowledge or pre-training.

Step 2: Local Model Training

Each client *k* trains a local model on its local dataset $D_{k}$. During training, clients perform a local update by minimizing the local loss function $L_{k}\left(\theta \right)$, which is defined for each client based on the local dataset. The update rule for the local model is

(2) $$\theta_{k}^{t+m}=\theta_{k}^{t}-\eta_{k}\nabla L_{k}\left(\theta_{k}^{t}\right)$$

(3) $$L_{k}=-\sum_{i=1}^{n}y_{i}\log\left({\hat{y}}_{i}\right)$$

where $\theta_{k}^{t}$ is the model parameters at the *t*-th iteration for client *k*, $\eta_{k}$ is the local learning rate, *m* is the aggregate that the client undergoes in every training of *m* epochs. $\nabla L_{k}\left(\theta_{k}^{t}\right)$ is the gradient of the local loss function. Each model is optimized by the local loss function defined in Equation (3), where *n* indicates the number of classification categories, $y_{i}$ represents the ground truth labels, and ${\hat{y}}_{i}$ denotes the model’s predicted probability distribution.

Step 3: Upload Local Model Updates to Central Server

After training locally for several iterations, each client *k* sends its updated model $\theta_{k}^{t+m}$ to the central server. The local update is computed as

(4) $$\Delta \theta_{k}=\theta_{k}^{t+m}-\theta_{k}^{t}$$

Step 4: Model Aggregation

The FedAvg algorithm is executed by the central server to integrate locally trained models from distributed clients. Through weighted combinations based on respective dataset sizes, an updated global model $\theta^{t+m}$ is produced. The weight for each client is typically based on the size of the local dataset $|D_{k}|$. The global model update is computed as

(5) $$\theta^{t+m}=\sum_{k=1}^{K}\frac{|D_{k}|}{\left|D\right|}\theta_{k}^{t+m}$$

where *K* is the number of clients, $|D_{k}|$ is the number of samples in the local dataset $D_{k}$, and |D| is the total number of samples across all clients.

Step 5: Global Model Update

Following the aggregation phase, the central server disseminates the newly optimized global parameters $\theta^{t+m}$ to all participating clients.

(6) $$\theta_{0}^{t+m}=\theta^{t+m}$$

Subsequently, all participating clients initialize their local models with the latest aggregated global parameters before commencing the next training iteration.

Step 6: Repeat Steps 2–5

The process of local model training, uploading updates, and aggregating the models continues for multiple rounds until the global model converges or the maximum number of communication rounds is reached.

Step 7: Evaluation

Upon completion of the training phase, the global model $\theta_{T}$ undergoes performance evaluation on a centralized test dataset by the server. The evaluation is typically carried out using a recognition accuracy metric.

### 3.4. Distance-Based Feature Selection Method

To boost the optimal representativeness and discriminability of features used in the federate fine-tuning phase, we propose a distance-based feature selection method, with its detailed implementation procedure described below. For the training dataset of a specific modulation type, high-dimensional feature representations are first extracted through a multi-channel feature extraction network and a temporal feature modeling architecture, denoted as $\varphi_{i}\in R^{d}$, where *d* represents the feature dimension, and *i* indicates the *i*-th modulation type. The subsequent processing involves two key steps:

Step 1: Feature Distribution Range Estimation

Calculate the statistical centroid for each feature class:

(7) $$u_{i}=\left(1/N\right)\sum_{j=1}^{N}\varphi_{i}^{j}$$

Here, *N* represents the number of samples for the given class, $u_{i}$ represents the feature center of the *i*-th modulation type, and b represents the feature obtained by a sample of the *i*-th modulation type through the feature extractor. The feature distribution boundaries $d_{\max}$ are determined using the maximum distance criterion as follows:

(8) $$d_{\max}=\max∥u_{i}-\varphi_{i}^{j}∥\left(1\leq j\leq N\right)$$

This value quantifies the distribution breadth of the current modulation type in the feature space.

Step 2: Typical Feature Selection

Based on a predetermined truncation threshold ε=0.5, a subset of typical features $\varphi_{\mathrm{typical}\;}$ is constructed:

(9) $$\varphi_{\mathrm{typical}\;}=∥\varphi_{i}-u_{i}∥<\varepsilon d_{\max}$$

The proposed method utilizes a nearest-neighbor sampling strategy to select the 100 most representative feature samples from $\varphi_{\mathrm{typical}}$. This selection process ensures the chosen samples are both tightly clustered around the class-conditional centroid in the feature space and sufficiently diverse to capture intra-class variations. By jointly optimizing for spatial density and distribution coverage, the algorithm constructs a compact yet discriminative subset that effectively characterizes the underlying feature distribution while mitigating sampling bias.

### 3.5. FedeAMR with Feature Fine-Tuning

As shown in Figure 1 and Algorithm 1, the proposed method incorporates a distance-based screening mechanism to identify and extract typical feature vectors for each modulation type during client-side local training. These representative feature vectors, which capture the most discriminative characteristics of the respective modulation schemes, are then transmitted to the central server alongside the locally trained model parameters. On the server side, global model aggregation is performed using the conventional FedAvg algorithm to integrate the distributed knowledge from all participating clients. To further mitigate the performance degradation caused by non-IID data distributions across clients, the server adaptively fine-tunes the classification layer by leveraging the aggregated representative feature vectors. This dual-phase optimization strategy, combining federated averaging for global parameter fusion and feature-guided fine-tuning for decision boundary refinement, effectively reduces the divergence in feature representations among heterogeneous clients, thereby enhancing the overall robustness and accuracy of the federated learning system.
**Algorithm 1** FedeAMR-CFF: 
**Input:** 
Client datasets $X_{1},X_{2},X_{3},$ and $X_{4}$ initialize the model $f_{0}$; 
**Output:** 
The trained federated learning model $f_{fed}$; 
**Step 1:** 
**Client Training:**(1)Each client performs local training using its dataset according to Equations (2) and (3); 
**Step 2:** 
**Distance-based Characteristic Feature Selection:**(1)Each client computes the feature centroids for each modulation type based on Equation (7);(2)Each client calculates the feature distribution range for each modulation type using Equation (8);(3)Each client selects qualified representative features according to Equation (9);**Step 3:** 
**Server Fine-tuning and Client Model Update**(1)Each client uploads its local parameters and the representative features selected in Step 2 to the server;(2)The server performs parameter aggregation based on Equation (5);(3)The server fine-tunes the aggregated model using the representative features;(4)The server distributes the fine-tuned model to all clients for parameter updates; 
**Step 4:** 
**Iteration**(1)Repeat **Steps 1–3** until model convergence or the specified number of training rounds is reached.

## 4. Experiment and Analysis

### 4.1. Training Details

The training set data is randomly partitioned into four distinct subsets, each serving as the training data for individual client nodes. Each client employs the Adam optimizer during local training iterations, with a learning rate of η= 0.0003 and a mini-batch size of |B| = 256, to prevent convergence to local optima. A total of 20 training cycles were performed, and in each cycle, the client performed five epoch trainings. For each round of server side fine-tuning, we froze the multi-channel feature extractor and temporal feature module, performing parameter fine-tuning solely on the fully connected classifier. The optimizer employed is Adam, where the learning rate is set to 0.0003, and the batch size is fixed at 100. All experiments were conducted in a Windows environment using the open-source PyTorch (0.3.0) framework for both training and testing, with an NVIDIA GeForce RTX 4060 (8 GB) GPU.

### 4.2. Evaluation Metrics

To comprehensively evaluate the classification performance of the model, the following six commonly used metrics were adopted in this study: Recognition accuracy, precision, recall, F1-score, sensitivity, and specificity. These metrics were calculated based on the true positives (TP: Correctly predicted positive samples), false positives (FP: Negative samples incorrectly predicted as positive), true negatives (TN: Correctly predicted negative samples), and false negatives (FN: Positive samples incorrectly predicted as negative) in the confusion matrix. Their definitions are as follows:

(1) **Recognition accuracy** measures the proportion of correctly classified samples out of all samples, reflecting the overall prediction performance.
  (10) $$\;\mathrm{Recognition}\;\mathrm{Accuracy}\;=\frac{TP+TN}{TP+TN+FP+FN}$$
(2) **Precision** represents the proportion of true positive samples among those predicted as positive.
  (11) $$\;\mathrm{Precision}\;=\frac{TP}{TP+FP}$$
  High precision indicates a low false positive rate, meaning the model’s positive predictions are highly reliable.
(3) **Recall** measures the proportion of actually positive samples that are correctly predicted as positive by the model. It reflects the model’s ability to comprehensively identify positives and minimize false negatives. A high recall denotes strong coverage of true positives with minimal missed detections.
  (12) $$\;\mathrm{Recall}\;=\frac{TP}{TP+FN}$$
(4) **F1-score** is the harmonic mean of precision and recall, balancing both metrics:
  (13) $$\;F1-\mathrm{score}\;=2\times \frac{\;\mathrm{Precision}\;\times \;\mathrm{Recall}\;}{\;\mathrm{Precision}\;+\;\mathrm{Recall}\;}$$
(5) **Specificity** denotes the model’s ability to correctly exclude negative samples, i.e., the portion of truly negative samples that are correctly rejected:
  (14) $$\;\mathrm{Specificity}\;=\frac{TN}{TN+FP}$$
  High specificity indicates that the model has few false positives.

### 4.3. Experimental Result

We evaluated the performance of the proposed federated learning method from three aspects: first, we provide a comparison with the results generated by local clients in Section 4.3.1. Second, we compare our federate method with the centralized training under data-access permission conditions in Section 4.3.2. Third, we detail the ablation studies for the proposed federated feature fine-tuning method in Section 4.3.3.

#### 4.3.1. Comparison with Local Client Result

**Figure 3:**
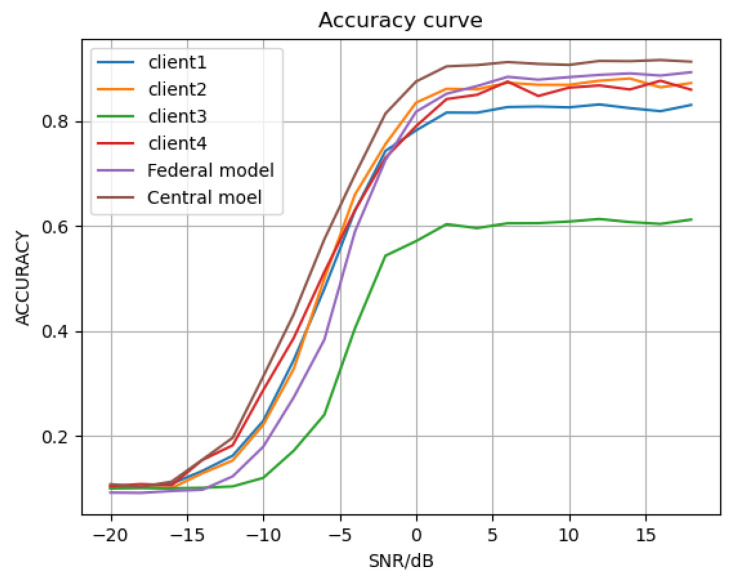
Recognition accuracy for each model under different SNRs.

The recognition accuracy of each client model and the federated model FedeAMR-CFF are shown in Table 1 and Figure 3. The experimental results demonstrate that the four locally trained models achieve accuracies of 50.93%, 53.03%, 36.20%, and 53.28%, respectively, exhibiting significant performance variations on the test set. The federated model aggregated via the proposed FedeAMR-CFF method attains 56.71%, outperforming all local client models. This discrepancy primarily stems from heterogeneous data distribution characteristics, particularly the client with merely 36.20% accuracy, which likely suffers from severe class distribution skew or data quality issues. The performance improvement of the federal model validates the advantage of our proposed framework in achieving knowledge complementarity and regularization effects through parameter aggregation and characteristic feature fine-tuning without the need for data from each client. When SNR is 12 dB, our FedeAMR-CFF method outperforms the best local client2 by 1.12%. When SNR is 18 dB, FedeAMR-CFF outperforms the best local client2 by 2.06%. These findings confirm that while data heterogeneity affects local model performance, our appropriate aggregation method can still realize collaborative gains. Although the centralized MCLDNN model achieved the highest accuracy of 61.18% with the usage of the client data, our proposed federated fine-tuning AMR can enhance classification performance without sharing or compromising any local client data.

As shown in Figure 3, with the increased SNR, the modulation recognition capability of different local clients improves. The proposed federated model effectively achieves both local parameter aggregation and central parameter fine-tuning, progressively approaching the upper-bound performance of the centralized model trained with shared data. These results further demonstrate that our method can perform effective parameter aggregation while strictly maintaining privacy preservation.

**Figure 4:**
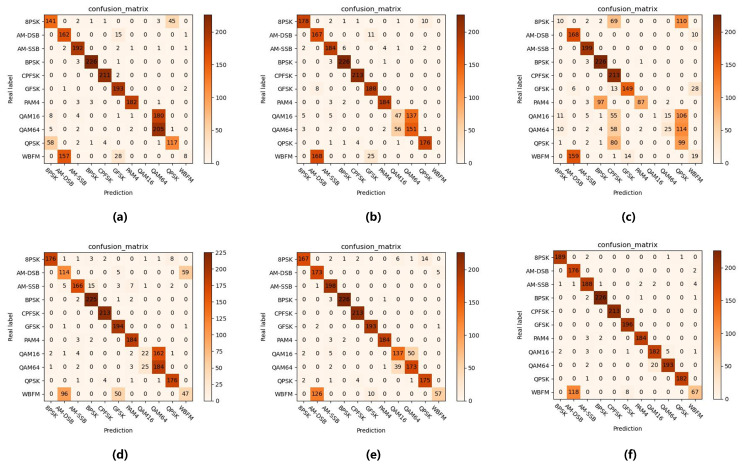
Confusion matrices at a 4 dB signal-to-noise ratio for all models: (**a**) Local Client1; (**b**) Local Client2; (**c**) Local Client3; (**d**) Local Client4; (**e**) Federal Model; (**f**) Central Model.

The confusion matrices of each model when SNR is 4 dB are shown in Figure 4. The model of Client1 exhibits significant inter-class confusion, misclassifying nearly all QAM16 signals as QAM64 while also misclassifying 81.34% of WBFM signals as AM-DSB. Additionally, there is a mutual misjudgment rate of approximately 28.53% between BPSK and QPSK. The models of Client 2 and Client 4 demonstrate similar error patterns, primarily characterized by partial confusion between QAM16 and QAM64, as well as misclassification between WBFM and AM-DSB. Notably, due to the poor quality of its training data distribution, the model of Client 3 shows significant confusion among almost all modulation types except AM-SSB, achieving an overall recognition accuracy of only 36.20%. The federated aggregation model exhibits a clear performance improvement, with its confusion mainly concentrated between QAM signals, showing an error rate of 22.30% and misclassifying 65.28% of WBFM signals as AM-DSB. In contrast, for the upper bound performance, the central model achieves optimal performance, only struggling with partial WBFM to AM-DSB misclassification and having slight difficulty in distinguishing some QAM16 and QAM64 signals, with an error rate of 6.25%.

As shown in Table 2, the federated model achieves 88.42% precision and 86.68% recall, representing significant improvements over the best-performing local model with 74.96% precision and 78.24% recall while approaching the performance of the central model at 92.66% precision and 91.33% recall. The 98.69% value for specificity indicates robust negative sample recognition capability, with only a 1.31% false positive rate. Notably, the F1-score of 85.61% reveals that FedeAMR-CFF maintains an optimal balance between precision and recall, outperforming the best Local Client4 by 10.65% while closely approaching the performance of the central model trained with full data.

This performance gain stems from three key technical innovations. First, the adaptive parameter aggregation technique effectively reduces variance among local models. Second, the proposed feature selection method successfully compensates for individual clients’ data bias. Third, the proposed FedeAMR-CFF framework effectively aggregates parameters from heterogeneous local models while preserving data privacy. The results conclusively validate that federated learning can bridge the performance gap between isolated local training and centralized training while completely avoiding raw data transmission and maintaining strict privacy preservation.

#### 4.3.2. Comparison with Centralized Full Data Training

**Figure 5:**
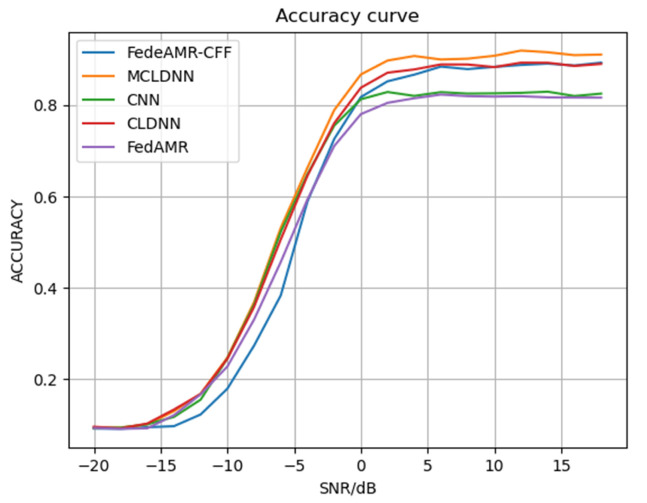
The comparison results between the proposed federated learning model and other methods.

As shown in Table 3 and Figure 5, the centralized CNN-based model with shared training data achieves 56.70% accuracy. The proposed FedeAMR-CFF achieves 56.71% accuracy in a parameter-only transmission setting with no data exchange. Additionally, the centralized CLDNN achieves an accuracy of 59.39%. The result achieved by our federated learning method is relatively close to it. Under the federated learning training conditions, our method outperforms FedeAMC by 6.12% at 6 dB and by 7.55% at 18 dB. Additionally, our method achieves an average performance improvement of 1.76% over FedeAMC. These results further confirm that the feature selection method and targeted fine-tuning method in FedeAMR-CFF can effectively enhance the federate learning capability of the model.

**Figure 6:**
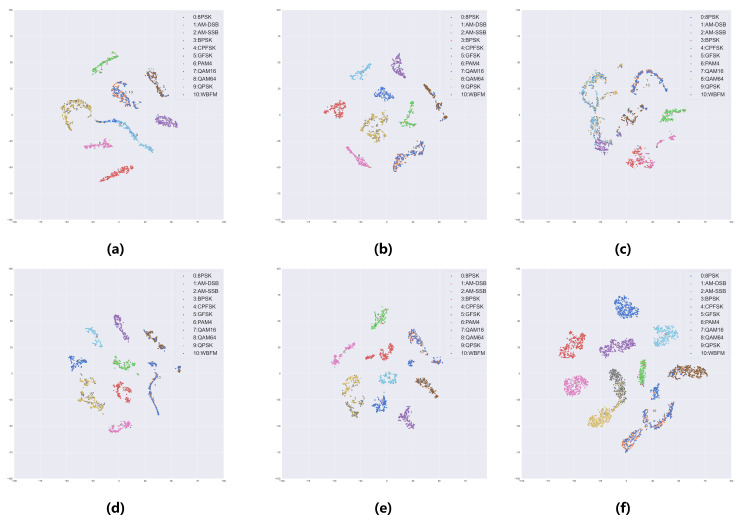
Visualization of t-SNE at a 4 dB signal-to-noise ratio for all models: (**a**) Local Client1; (**b**) Local Client2; (**c**) Local Client3; (**d**) Local Client4; (**e**) Federal Model; (**f**) Central Model.

The feature visualization results of each model are shown in Figure 6. It can be observed that in the Client 1 model from subplot (a), QAM16 and QAM64 samples exhibit extensive overlap in the embedding space, while WBFM and AM-DSB samples also demonstrate mixed distribution patterns. These findings are highly consistent with the error patterns observed in the confusion matrix. The feature space of Client 3 in subplot (c) exhibits pronounced class confusion, with only AM-SSB signals forming distinct clusters. The federated aggregation model feature space displays good inter-class separation, with only partial overlapping regions remaining among QAM signals as well as between WBFM and AM-DSB. The central model’s feature space presents the clearest class boundaries with the shared data, forming compact and well-separated clusters for most signal types, except for some overlap between WBFM and AM-DSB. These visualization results provide intuitive evidence of the critical impact of data distribution quality on model discriminative capability. They also demonstrate that federated learning can effectively integrate feature learning advantages from different clients, achieving performance approaching centralized training while maintaining data privacy protection.

#### 4.3.3. Ablation Study for Feature Fine-Tuning

The experimental results in the first row demonstrate that the optimal result generated by the local client during training is 53.28%. The performance in the second row represents the effect of the central server using FeaAvg in federated learning. It can be seen that using FedAvg has improved the result by 1.66%. After introducing the feature-fine-tuning method proposed in this paper, the result has been improved by 3.43% compared with the best result generated by the local client. As demonstrated in Table 4, the aggregated model employing typical feature fine-tuning achieves an overall accuracy of 56.71% on the test set, with an improvement of 2.17% over the baseline FedAvg. These findings demonstrate that our method effectively enhances the recognition performance of the federated aggregation model through the design of a typical feature fine-tuning mechanism.

## 5. Conclusions

This study innovatively proposes a federated learning framework named FedeAMR-CFF, which significantly enhances modulation recognition performance while ensuring data privacy security. Specifically, the FedeAMR-CFF framework incorporates the following key innovations: (1) During the local training phase, a distance-based feature selection mechanism is adopted to extract the most representative feature vectors for each modulation signal category. (2) In the server aggregation phase, it creatively combines model parameter aggregation with the feature fine-tuning strategy—integrating global model parameters through the FedAvg algorithm while optimizing the fully connected classifier using selected characteristic features. This dual optimization mechanism not only achieves efficient cross-client knowledge fusion but also effectively addresses challenges posed by non-IID data. The experimental results on public datasets demonstrate that the FedeAMR-CFF framework achieves a 3.43% recognition accuracy improvement over the best-performing local model while strictly safeguarding data privacy security for all participating parties. This significant performance enhancement validates the effectiveness of our approach in federated learning environments.

## Figures and Tables

**Table 1:** The recognition accuracy of each signal-to-noise ratio and the overall recognition accuracy of each model.

SNR (dB)	Local Client1	Local Client2	Local Client3	Local Client4	FedeAMR-CFF	Central Model
−18	10.24%	10.22%	10.04%	10.80%	9.13%	10.29%
−12	16.22%	15.30%	10.36%	18.19%	12.27%	19.64%
−6	48.03%	49.99%	24.02%	51.22%	38.34%	57.48%
0	78.22%	83.51%	57.11%	79.02%	81.78%	87.54%
6	82.68%	87.31%	60.52%	87.53%	88.44%	91.26%
12	83.17%	87.69%	61.34%	86.79%	88.81%	91.47%
18	83.07%	87.25%	61.22%	85.99%	89.31%	91.32%
All	**50.93%**	**53.03%**	**36.20%**	**53.28%**	**56.71%**	**61.18%**

**Table 2:** The model’s performance on precision, recall, specificity, and F1-score when SNR is 4 dB.

Metric	Local Client1	Local Client2	Local Client3	Local Client4	FedeAMR-CFF	Central Model
Precision	70.28%	72.63%	62.69%	74.96%	88.42%	92.66%
Recall	74.18%	78.24%	54.17%	77.29%	86.68%	91.33%
Specificity	97.50%	97.85%	95.48%	97.79%	98.69%	99.14%
F1-score	68.49%	74.36%	47.74%	74.96%	85.61%	90.44%

**Table 3:** The comparison results between the federated learning model and other methods.

SNR (dB)	FedeAMR- CFF	Other Model			
		MCLDNN [31]	CNN [25]	CLDNN [30]	FedeAMC [17]
−18	9.13%	10.29%	9.37%	9.22%	9.13%
−12	12.27%	19.64%	15.52%	16.76%	16.69%
−6	38.34%	57.48%	52.26%	50.60%	45.66%
0	81.78%	87.54%	81.29%	83.82%	78.03%
6	88.44%	91.26%	82.85%	88.92%	82.32%
12	88.81%	91.47%	82.95%	89.30%	81.96%
18	89.31%	91.32%	82.00%	88.59%	81.76%
All	**56.71%**	**61.18%**	**56.70%**	**59.39%**	**54.95%**

**Table 4:** Ablation test results.

Fedavg	Feature Fine-Tuning	Accuracy
×	×	53.28%
✓	×	54.54%
✓	✓	56.71%

## Data Availability

The original data in RML.2016.10A presented in this study are openly available at https://pubs.gnuradio.org/index.php/grcon/article/view/11 (accessed on 23 June 2025).
